# Trauma‐informed care in early intervention: Providers’ barriers, boundaries, and needs

**DOI:** 10.1002/imhj.70075

**Published:** 2026-01-18

**Authors:** Crystal S. Williams, Mia Chudzik, Emily E. Holden

**Affiliations:** ^1^ Department of Special Education University of Illinois Urbana‐Champaign Illinois USA; ^2^ Department of Special Education Department of Curriculum & Instruction University of Illinois Urbana‐Champaign Illinois USA; ^3^ School of Education and Human Development University of Colorado at Denver Denver Colorado USA

**Keywords:** early intervention, qualitative methods, trauma‐informed care

## Abstract

Young children who receive early intervention (EI) services are at increased likelihood of having experienced trauma, as trauma is most common in the birth‐to‐three age range, especially for children with disabilities. Little is known about EI services for trauma‐impacted children and families. Previous research suggested that EI providers are unprepared to support trauma. Thus, we conducted qualitative interviews with 14 EI providers in one US state to understand the barriers and needs related to trauma‐informed care (TIC). We organized our findings so that each theme is nested in a system from social ecological theory. The six themes included macrosystem (the system is unequipped, lack of role clarity), exosystem (providers need specific training), mesosystem (gatekeeping, family‐provider dynamics), and microsystem (EI providers’ well‐being and comfort). Implications include the need for a top‐down approach to TIC in EI and clarification of the roles of various EI disciplines in implementing TIC.

## 1 INTRODUCTION

Young children aged birth‐to‐three with disabilities and delays, or who are at risk for delays, can receive developmental services under Part C of the Individuals with Disabilities Education Act (IDEA, 2004), known as early intervention (EI). A portion of the children who receive EI services have likely experienced trauma. Trauma is conceptualized as an individual's negative response to a harmful event or experience that causes long‐lasting impacts to the individual (Substance Abuse and Mental Health Services Administration [SAMHSA], 2023). The traumatic event can include a myriad of experiences, including abuse, neglect, removal from a parent, witnessing violence, or systemic oppression (e.g., racism, ableism, classism; Goldin et al., 2023; SAMHSA, 2023). Children in EI are at an increased risk of having experienced trauma because it is most common in infants and toddlers (U.S. Department of Health and Human Services, 2025) and is even more prevalent in children with disabilities (Jones et al., 2012). Infants and toddlers who experience trauma may be impacted in the areas of attachment, cognitive development, and physical development (Melville, 2017). In some states (e.g., Ohio, Illinois), children who are the subject of an indicated allegation of abuse or neglect are automatically referred to or qualify for EI services (Children & Family Services Act, 2022; Ohio Admin, 2024). In fact, in 2023, over 80,000 children who had experienced trauma were eligible for EI services across the 38 US states that report this data (U.S. Department of Health and Human Services, 2025). Beyond children, caregivers in EI also may have experienced trauma. For example, EI providers said they believe caregivers in EI often have experienced trauma (Chudzik et al., in press). Given that EI services extend to children and families who have experienced trauma, it is important that the EI providers are equipped to support this unique population of children.

**Key Findings**

- Early intervention providers experience many barriers to trauma‐informed care, ranging from large systemic issues (e.g., provider shortage) to individual challenges (e.g., provider safety).
- There are inconsistencies in providers’ beliefs about the roles of various disciplines in providing trauma‐informed care, indicating a need for better clarity in the field.
- Early intervention providers need more targeted training on trauma‐informed care that incorporates reflection, practice, and learning from people with lived experiences.

**Statement of Relevance**

Young children with disabilities are likely to experience trauma, which impacts their social‐emotional development and mental health. Understanding the barriers and needs of early intervention providers to use trauma‐informed care is an important step in improving infant and early childhood mental health in the United States, as these providers are often “first responders” to families. Findings from our study can be used to improve early intervention provider training and develop systemic policies so that young children and their families who have experienced trauma are better supported by the system.

### 1.1 Research on EI and trauma

Some researchers have explored EI providers' experiences working with families in EI who have experienced traumatic events, such as abuse, homelessness, and poverty (Corr et al., 2019; Corr & Santos, 2018; Hurley et al., 2018; Spence et al., 2022, 2023; Williams et al., 2025). Some of these studies focused on a specific type of trauma. For example, Corr et al. (2019) conducted focus groups with EI providers to understand their experiences working with children who had experienced abuse or neglect and Hurley et al. (2018) interviewed providers about working with families experiencing homelessness. Using a broader approach, Spence and colleagues (2022, 2023) conducted focus groups with EI providers to understand their experiences implementing family‐centered practices with families experiencing “vulnerable circumstances,” including poverty, homelessness, substance exposure, foster care, and caregivers with a disability, though the authors did not refer to these circumstances as “trauma.”

Overall, existing research suggests that EI providers experience challenges supporting children and families who have experienced traumatic events. Some of these challenges included being uncertain about the signs of abuse and neglect and when to report their concerns (Corr et al., 2019), difficulty implementing EI recommended practices due to families’ complex circumstances (Corr et al., 2019; Hurley et al., 2018; Williams et al., 2025), difficulty scheduling and maintaining appointments (Hurley et al., 2018), not receiving preparation or training about abuse and neglect (Corr et al., 2019), and assuming personal risks and an emotional toll from their work with this population (Corr et al., 2019; Hurley et al., 2018; Williams et al., 2025). In addition to these challenges, researchers also found that providers have negative perceptions about families and their circumstances (Spence et al., 2022; Williams et al., 2025) and that professional collaboration for these families is lacking (Corr et al., 2019; Williams et al., 2025).

### 1.2 Need for trauma‐informed care in EI

While the research on trauma in the EI field is in its infancy, there is growing support and advocacy for trauma‐informed care (TIC) in EI (Blanchard et al., 2021; Munger et al., 2023). TIC is a strengths‐based and relationship‐centered approach that, when used by early childhood professionals, focuses on (1) recognizing that trauma impacts a child's development and behavior and (2) responding in a way that aims to reduce, rather than perpetuate, harm (Nicholson et al., 2023). Munger et al. suggested that TIC in EI is needed to help bridge the gaps between EI and the child welfare system, which serves many trauma‐impacted children and families. The authors advocated for increased training and preparation for EI providers to focus on TIC. Additionally, TIC also has been viewed as a way to respond to systemic oppression, such as ableism and racism, toward more justice‐oriented services for children and families (Blanchard et al., 2021; Venet, 2021). Thus, understanding how TIC is used in EI as well as the barriers and needs of providers to use this approach is essential.

This study is part of a larger qualitative project exploring EI providers’ beliefs and experiences with TIC (Chudzik et al., in press). For this particular paper, we present data related to participants’ beliefs about barriers and needs related to TIC. To learn more about providers’ definition of trauma and trauma‐informed care as well as their trauma‐informed practices, refer to Chudzik (in press). The research questions that guided this study were: (1) What are the barriers to using TIC in EI? (2) What do EI providers need to implement TIC in EI?

### 1.3 Theoretical framework

We used Bronfenbrenner's social ecological theory (1977) to guide the analysis of this study. This theory suggests that multiple intersecting environments and systems impact child development. After our initial open‐ended coding, we developed broad themes that we organized by the social ecological systems levels (i.e., microsystem, exosystem, mesosystem, and macrosystem). We decided to do this intentionally because multi‐level factors emerged during analysis; thus, this theory helped us articulate factors across levels that impacted providers’ use of TIC. In this paper, we used this theory to frame our findings and discussion.

## 2 METHODS

To gain insight into the barriers of TIC for EI providers, we used a qualitative research design, consisting of semi‐structured interviews. Our approach was constructivist in nature, as we positioned EI providers as experts on their experiences (Creswell & Creswell, 2018).

### 2.1 Positionality

Positionality is an acknowledgement of how researchers’ identities, backgrounds, experiences, and values influence the research process (QR Collective, 2023). Our research team consisted of three White women, two of whom were postdoctoral researchers and one of whom was a doctoral student. The first author's primary research focus is on EI services for families of children with medical complexities, while the other two authors focus on TIC in early childhood settings. Our combined interests motivated us to explore TIC in EI. Across our team, we also have representation from a former early childhood special education teacher, former EI providers, a current school psychologist, and parents of young children. Our collective backgrounds were imperative for designing, conducting, and analyzing the study. For example, the first author's experience as a provider in the state allowed her to gain access to closed social media groups used for recruitment. Additionally, the recruiting authors’ positions as White women may have contributed to our participant sample also representing only White women. Finally, our knowledge and experience with EI in the state were helpful for building rapport with participants during interviews and understanding the nuances of the data in relation to the state.

### 2.2 Participants

Fourteen EI providers in Illinois participated, including six speech‐language pathologists (SLPs), three developmental therapists (DTs), two occupational therapists (OTs), one physical therapist (OT), one social worker/psychologist (SW), and one provider who identified as both SW and DT. All 14 participants identified as female and 13 identified as White (one did not provide race/ethicity information). They had worked in the EI field for an average of 15.4 years (range = 7–38) and had an average caseload of 22.7 families (range = 7–55). Participants selected or were assigned a pseudonym, depending on their preference, which is used throughout the findings.

### 2.3 Data collection tool

The first and second authors developed the semi‐structured interview in collaboration, combining their knowledge and expertise of EI and TIC. We piloted the protocol with an EI provider in Colorado who had experience working with trauma‐impacted families. She provided feedback that we used to improve the interview protocol and procedures, such as changing the wording of some questions and sending participants the questions ahead of time. The final protocol had 16 main questions and additional probes (see Chudzik et al., in press for the protocol). The questions covered a variety of topics, including trauma, TIC, training experiences, and barriers and facilitators of TIC. The interviews were estimated to take around 60 min.

### 2.4 Data collection procedures

This study was approved by the Institutional Review Board in April 2024. We began recruiting participants in May 2024. We shared the study information by posting our study flyer in EI‐related social media groups. Participants completed an online survey to indicate their interest in the interview and to ensure they met the screening criteria. We were looking for EI providers in Illinois who provided ongoing services to families and had worked with at least one family who they believed had experienced trauma. The participants did not have to have previous knowledge or experience with TIC to participate. Once they confirmed they met the eligibility requirements, the survey directed them to an online consent form. If they agreed to participate, the survey then prompted them to sign up for a Zoom interview and answer demographic questions.

The first and second authors conducted the first four interviews together. The remaining 10 were conducted independently by the first or second author. The interviews ranged from 23 to 60 min (*M* = 50), amounting to 704 min of interview data across participants. Interviews were recorded and transcribed using Zoom. The first and second authors wrote reflexive memos after each interview they conducted. They also conducted member checks with participants. To do this, the first or second author reviewed the initial transcript as soon as possible after the interview and wrote a one‐page summary of what was learned during the interview. Then, the second author invited participants to correct, clarify, or confirm their summary for accuracy. Only one participant made minor edits, while the rest confirmed it was accurate.

### 2.5 Analysis

To prepare the data for analysis, the third author reviewed the transcripts against the audio recordings, corrected them for errors, and replaced the participants’ names with their selected pseudonyms. Then, all three authors uploaded the corrected transcripts to MAXQDA (Verbi Software, 2024), a qualitative data analysis software, to analyze the data. Beginning with the first interview transcript, all three authors independently coded the transcripts using descriptive, open‐ended coding procedures, in which the codes were developed from the data (Saldaña, 2021). We met as a team to discuss and agree on codes for each transcript. During the first meeting, we developed a codebook in MAXQDA, which featured our agreed‐upon codes and brief definitions of each code, which served as the basis for coding the remaining interviews. The codes were sorted in the codebook by major topics, such as Barriers, Practices, and Needs. Additional codes were added throughout the analysis as needed to capture novel information. When we made changes to the codebook, the second author reviewed previously coded data to ensure that it was accurately represented. We reached consensus on all coded passages for each transcript, which was tracked by the second author and then sent to the others after each meeting.

After this initial coding phase was finished for all interviews, we collaboratively searched for patterns, or themes of the data (Saldaña, 2021). For this paper, each author reviewed the agreed‐upon codes and coded data related to Barriers and Needs to develop initial themes. Each author filled out a spreadsheet with their initial themes, explanations of themes, supporting codes, and sample quotes. Together, we combined, separated, and reorganized our initial themes until all authors’ ideas were captured, resulting in six agreed‐upon themes.

### 2.6 Credibility

To ensure the quality of our study and analysis, we used credibility and trustworthiness measures for qualitative research in special education (Brantlinger et al., 2005; QR Collective, 2023). We demonstrated reflexivity throughout the study, such as writing memos about our perceptions of the data, engaging in reflective conversations, and reporting our positionality in this paper. We also engaged in collaborative work, such as having multiple researchers develop the interview questions, inviting an EI provider to give feedback on the interview protocol, and using multiple researchers to analyze the data. Another credibility measure used was member checking, in which participants reviewed a summary of their interview to ensure their data was accurately represented. Finally, we provide rich descriptions of our themes using many participant quotes as evidence of our conclusions.

## 3 FINDINGS

The themes are organized by the systems of social ecological theory (Bronfenbrenner, 1977). First, we give a brief description of each system and then describe the themes that fall under each system. We begin with the largest system and end with the smallest.

### 3.1 Macrosystem

The macrosystem is the established society and culture in which a child develops, including policies, norms, and values. We identified two themes in the macrosystem: the system is not equipped to support TIC and there is a lack of role clarity for TIC in EI.

#### 3.1.1 The system is not equipped to support TIC

Broad issues in EI and related systems were reported as a barrier to using TIC. Participants noted that the EI system itself was not structured in a way that promoted TIC. They cited the lack of providers and cohesive teaming, untimely procedures, unbillable hours of work, and arbitrary rules as limiting their use of TIC. Participants believed that there were not enough providers to support TIC in EI. Sometimes, families waited a long time for services, contributing to distrust in services. For example, Lucia (SLP) explained,

Sometimes I go into a home, and they're like, “We waited 3 months for you to call us!” And I'm like, “Well, I didn't know about you until Tuesday.” So I feel like, they want to be mad at being abandoned or like forgotten about, but that's not what happened.

Willow (DT) gave another example of how long it takes to obtain services:

It takes a long time to get anything done. So for example, that family that needs the mental health provider, I think I called that coordinator, maybe 3 weeks ago, and I still haven't heard if we've had an evaluation done to get that service. And that's not to say anything bad about people that are working in EI, that's just the process, right?

Participants also critiqued arbitrary rules in EI, like enhanced eligibility, as contributing to the lack of providers. For example, Emma (SLP) critiqued a new rule in Illinois that allows children with summer birthdays to continue receiving services until August, as it has resulted in a greater shortage of providers and longer wait times. She explained, “They just make these random rules without putting programming in place to make sure that it happens.”

The lack of SWs specifically was a concern that providers believed inhibited TIC in EI. Sarah (SLP) explained, “We have families that have experienced trauma or are going through traumas, but we don't have the social workers to support… There's like no hope at all in getting a social worker to even do monthly check‐ins.” Terry (OT) elaborated on this issue, explaining that even when SWs were available, they were not always experienced in TIC. She explained,

There certainly are not enough social workers and there are not enough social workers who are specifically trained or interested in trauma informed care. We tend to have a couple who really just want to do resources. They're really good at getting that family a new Medicaid card or getting them signed up for social security or getting them transportation to a doctor's office. But that's where they want to leave it.

Participants also reported that the EI system did not support effective teaming with families or providers, which they felt was important for TIC. Charlotte (SLP) discussed how difficult it was to support trauma‐impacted families because their needs sometimes go beyond what she can bill for. She explained,

We don't get paid at all for any time we're spending outside of the session, pretty much unless it's [Individualized Family Service Plan] billable time. But if I'm talking to a family a couple of days later, to check back in or whatever, I can't bill for any of that time. I think that is hard…I'm not gonna *not* connect with the family outside of session if it feels important, but I do feel like I do limit it.

Speaking about the barrier of teaming with other professionals, Kylan (PT) expressed,

I wish that there were more cohesive teams and the communication between teams was easier and kind of built into the structure of it a little bit more. The way that it is now. It's just so, willy‐nilly. And it's like you have to be in the CIA to even figure out who are the other people on the team.

Shirley (SLP) shared a different concern about teaming, saying: “I find it might be hard for families who have a trust issue based on trauma, whatever it is, to have so many people to all of a sudden trust.” In this quote, Shirley believed that the EI system's practice of assigning multiple providers to a family may not work for those who have experienced trauma.

Providers also believed that a barrier to TIC was connecting families to other systems, services, or resources outside of EI. For example, Elizabeth (SW/DT) explained the challenge of finding quality mental health providers that accept Medicaid. She said, “When a family has experienced a lot of trauma…the limitation is often with the medical card. It's much harder to find mental health [services] without a huge wait list or without somebody that might be a student.” Elizabeth further elaborated on how this is particularly an issue when children exit EI, sharing, “They graduate from early intervention, and especially the families who are on Medicaid, there's no one to pass them on to. I tried. I get no calls back. The kids are falling in the cracks.” She also reported worrying about the future of these children, showing some negative perceptions about their futures. She explained, “I'm envisioning certain kids shooting people from a clock tower. You know what I mean. You look at these kids, like these kids need help, and you can't find it.” Participants also reported a lack of resources to help families meet their basic needs. For example, Kylan (PT) reported, “There's a lot of communities that have a lot of barriers to a lot of different things, from barriers to healthy food, and barriers to medical, quality medical care, safe and affordable housing, and there's so many things.” In sum, systemic issues in both EI and related systems were considered barriers to using TIC in EI.

#### 3.1.2 Lack of role clarity related to TIC

The lack of role clarity about TIC within the EI system impacted providers’ use of TIC. Some participants were unclear about if, how, and to what extent TIC fit into their role as an EI provider. For example, Clover (SLP) described being unsure if TIC fell into her scope of practice, saying:

There's definitely that, “What is my scope of practice?” I like to feel like I'm a trauma‐informed speech therapist. But I think you just go back and forth in your head. Because I think in school it's really drilled into you, “You're gonna wear a lot of different hats”… but then there's our scope of practice through [American Speech‐Language‐ Hearing Association], the national organization. Well, technically, this is kind of in our scope of practice, kind of not, but I don't know.

Similarly, Kylan (PT) shared, “Certainly there's a lot of TIC that is outside of my scope of practice. And so I think that is also something that could be kind of tricky in EI is that you get pulled in for all sorts of different things…But it's challenging, because it's not really, my– I'm here to do physical therapy.”

Some participants described a need for all EI providers to use TIC, regardless of their specific role. Terry (OT) described this by saying, “I think other disciplines don't think it's their job. The speech therapist is like, ‘Well, that's not me. We've got to get the social worker involved.’ I think all of us as humans, it's our job.” Willow (DT) believed it was within her role to provide mental health support given the context of where she works. She explained, “In early intervention, every discipline, I feel like, is a mental health provider. Especially again down here [Southern Illinois], we use developmental therapists as kind of placeholders for other [disciplines] that we don't have access to down here.” Some participants, like Elizabeth (SW/DT), wanted all EI providers to use TIC, but recognized the limitations with this, sharing: “I would love for everybody to have some education and training on trauma‐informed strategies. But I think that it's almost outside of their scope to really know a lot of the specifics, or to know exactly what to do.” Other participants felt that some EI providers were overstepping their roles. Penelope (SW) gave an example of this problem, sharing:

For some reason they don't want psych/counseling providers involved, and I don't know what those dynamics are…but that's a concern to me. If other team members think, “Well, we went to a trauma‐informed training so we're fine, we don't need a clinician.” That's not good practice. That's not fair to children or families.

Shirley (SLP) also had concerns about EI providers going out of their scope of practice when trying to use TIC, saying, “I also think I've seen providers who go too far with a family, and they don't have a skill set for it. And that troubles me in many respects.” Overall, this lack of clarity in the EI system regarding who should use TIC and in what way led to challenges for participants.

### 3.2 Exosystem

The exosystem is the indirect environmental factors that impact a child, including the support systems that their caregivers and EI providers receive. We identified one theme related to the exosystem that is needed to improve TIC in EI: Training specific to TIC in EI.

#### 3.2.1 Providers need training specific to TIC in EI

A significant barrier to quality implementation of TIC in EI was a lack of adequate training. Kylan (PT) and Clover (SLP) both cited a lack of information about TIC in EI as a reason for everyone in the system being “on a different page” about TIC. A common complaint was that available trainings were too basic and focused on definitions rather than practice or application. Elizabeth (SW/DT) described a training she attended:

It was more, “This is what trauma is. This is what a child might be exhibiting. These behaviors might come from this.” But nothing about, “What do we do about it? What advice can we give the parents?” That's where the limitations with the early intervention trauma trainings are is that they stop at anything that would make you [be] helpful to the family in regards to the trauma.

Emma (SLP) also felt that trainings offered by EI were too basic. She shared, “They're not really fruitful if you've been in EI for more than like five or six years.” Scarlett (DT) noted that the one training she received was “just a lot of stuff that you would know just as a person living in this world.”

Participants had many ideas for how training opportunities could be modified to better meet their needs. They expressed needing training to be more application‐focused, role‐specific, accessible, and ongoing. Kylan (PT) captured the key element of training practitioners desired the most: practical application: “It can be sort of overwhelming, like trauma‐informed care sounds great but you're like, ‘What does that mean on an individual case by case basis?’” Elizabeth (SW/DT) elaborated on this, wanting clarity around application of TIC specific to EI:

I would love to have gotten some trauma training as it directly related to early intervention and the role of it in early intervention…we kind of have to wonder, “When does it become outside of the scope of early intervention from a developmental standpoint?”

Providers like Kylan (PT) and Kimberly (OT) requested an even more narrow application focus, hoping for training focused on their specific discipline. Kimberly (OT) shared, “I wish that there was more out there on how to implement it. I haven't seen a course out there like trauma‐informed practice for occupational therapists, right? It's very much in the mental health fields.” She also pointed out that because current TIC trainings are not OT‐specific, she does not receive continuing education credit for them, which disincentivizes her from taking them. One outcome of role‐specific TIC training that participants hoped for was clarity around the scope of their role. Charlotte (SLP) wanted to know when she should pull someone else in. She expressed, “I think there's a lot that I don't know. Maybe not things that I think I'm capable of doing myself, or that I could handle on my own, versus like this is not a choice [for me to address on my own].”

In addition to desires about the content of trainings, participants also shared hopes for the structure and approach to how TIC trainings would be delivered. Shirley (SLP) summarized, “It needs to be interactive. It needs to be over a period of time. It can't just be hit and run.” Terry (OT) addressed the interactive element, describing this possibility:

Watching somebody interact throughout a session where somebody has experienced a significant trauma. And, “Oh, that was really good when they did that,” or “I think they missed something there. Mom said this, and there was no follow up.”… So watching and doing a little critique. But then getting to practice those skills.

Charlotte (SLP) highlighted the importance of ongoing learning by commenting that, “It's hard to say like ‘I took a three hour course on being trauma‐informed and now I can check the box that I'm always 100% of the time trauma‐informed.’”

Lastly, some participants mentioned the need to ensure any TIC trainings were centering the experiences of those impacted by this work. Emma (SLP) shared that she would appreciate it if TIC trainings addressed intersectionality and “how I can best support families who might come from different backgrounds, have different experiences. Make sure that the care that I'm providing is beneficial and not offending anyone, or is inadvertently harmful.” Similarly, Clover (SLP) shared that she would like to hear the perspectives of adults who received EI services as children. These examples reinforce the overall theme of providers wanting to ensure that training opportunities prepare them to engage in meaningful, practical, TIC that supports the children and families on their caseload.

### 3.3 Mesosystem

The mesosystem is the interactions between individuals and environments that comprise a child's microsystem, such as caregiver–provider interactions or provider–provider interactions. We identified two themes within the mesosystem that inhibited providers from using TIC: gatekeeping and family–provider interactions and dynamics.

#### 3.3.1 Gatekeeping

At the mesosystem level, gatekeeping was found to be a barrier, in which access to TIC was inhibited by a lack of information or resources. One way gatekeeping prevented TIC was when services were withheld or difficult to access. This often happened with social work services, as participants felt like it was hard for families to get the service or add it later on. Families needing to disclose their trauma to justify the need for social work services was also perceived as a gatekeeping issue. Emma (SLP) described her concerns about this, saying:

A lot of families should be getting social work services through EI. And the role of social work, I feel like is not well defined or not well understood in EI. In general, you really have to justify need. And does that mean the family should have to disclose their trauma to people they don't know? Possibly, possibly not.

Kylan (PT) described another problem with social work services when she shared,

Usually social work is added later, it's not really the first line of defense. And so I sometimes feel like that is maybe a problem, because it's only after you're like “Yikes, this is crazy,” or something really bad is happening to call the social worker, and I feel like that is kind of doing them a disservice.

Lucia (SLP) proposed a solution to these issues, saying that social work services should be automatically given if there is any indication that trauma has occurred, that way “social work would be on board with the option of getting off, rather than taking so long to get on board and then so much happens that by the time the social worker gets on board they don't even know where to start.”

Some participants felt that those who did initial evaluations for EI served as gatekeepers for getting helpful services for families. Penelope (SW) shared a story about this:

A service coordinator recommended a social‐emotional evaluation for a family because of a history of trauma and the other two team members who were doing the eligibility evaluation said, ‘Oh no, we don't think they need that,’ and that is very troubling. That should not be the way this happens.

Initial evaluators also were seen as gatekeepers when they withheld important information from other providers. Some participants wished they could be a part of the initial evaluation or get more information from the evaluators so they could learn more about the child and family's background. Elizabeth (SW/DT), who serves as an initial evaluator, described the difficulty of balancing family privacy and sharing information with EI providers, saying, “it kind of goes through my mind, ‘How do we get everybody on the same page without revealing really private information that the family may have only shared with me?’”

Other participants focused on service coordinators specifically as gatekeepers. They recognized that service coordinators were often overwhelmed and had a lot on their plate, but also felt that they did things that prevented TIC from being used effectively. Kylan (PT) felt that service coordinators could do more to learn information about a family's background that could then lead to better TIC, saying:

They're not trained in social work or anything else. But sometimes the list of information that they ask is so specific in some ways, and so unimportant in other ways…But they never ask anything about the bigger things like, “What are you worried about?” That would kind of cue in if somebody needed more, if a family needed more support.

Lucia (SLP) also described some issues with service coordinators as gatekeepers of important information. She shared instances of not being told when a child or family was involved with the Department of Child and Family Services. She recognized that it did not have anything to do directly with speech services, but also felt that it was important to know what was going on with a family. She said, “The service coordinator will say, ‘I wasn't supposed to tell you,’ or ‘It's not my position to tell you,’ and I get that it doesn't have anything to do with speech, but it has so much to do with understanding what's going on.” Overall, participants felt that gatekeeping of seemingly relevant information and services prevented TIC from being used.

#### 3.3.2 Family–provider dynamics and interactions

The second meso‐level challenge to implementing TIC was related to family–provider dynamics and interactions. Participants believed that their ability to implement TIC was impacted by the ways families “showed up” for EI. For example, Emma (SLP) explained,

Many of the families were super duper, open, and eager to receive support … Some of them are really not. And for whatever reason, just not as available, either physically it's hard to schedule and get consistent attendance, or mentally and emotionally or culturally. Just where the families, they've experienced trauma, but they're not as available (not necessarily to disclose their trauma), but to receive support and to ask questions, and to ask for things that they might need or have questions about.

Similar to Emma's comment that some families were not available to receive support, participants also said that EI can be overwhelming or may not be the highest priority for families experiencing trauma. Charlotte (SLP) noted:

I think prioritizing therapy in general is also like‐ that's a barrier in that. If there's bigger, harder stuff going on then, of course I don't think you need to be prioritizing therapy the most of everything. But that said, how can we get this balance of some sessions without it feeling like it's too overwhelming?

Kimberly (OT) said that the trauma that families experienced can be a barrier to TIC, sharing:

A huge [barrier] is how the trauma is impacting the family, right? Because we know that especially in early intervention, again, the model is parent coaching. But if a parent has experienced trauma and is kind of shut down and doesn't want to be a part of it, you can't really cause growth, or help them to move forward. So the barrier to using trauma informed care is the trauma.

One participant, Penelope (SW), discussed a fine line between acknowledging how family dynamics prevent TIC and becoming frustrated with families because of it. She explained,

There are quite a few families over the years that our practitioners experience as frustrating and I think not always, but often, trauma can be related to that, and that's a key piece. It's not something that goes from the practitioner, necessarily towards the client, but for practitioners of any discipline to be aware that if a family is being frustrating, there's probably more dynamics going on.

Similarly, Kylan (PT) shared that sometimes, her feelings about the trauma go beyond her ability to maintain effective TIC. She explained, “If I feel like the child is suffering, it's hard for me to be non‐judgmental. So sometimes I'm judgmental. So that gets in the way.”

Participants also believed that families’ previous experiences or backgrounds sometimes prevented providers from effectively using TIC. For example, Lucia (SLP) reflected on how difficult it can be to build relationships with families and children if the caregivers do not trust the system. She explained,

There are some families who have been through so much trauma, and who have been so abandoned and hurt and jaded by the systems, early intervention being a system, that I feel like we don't get anywhere in our relationship, and I feel like it also affects the child. I feel like the child can't bond with me because their mom doesn't, you know?

Social work was another system that families particularly distrusted, according to participants. They explained that it was difficult to implement TIC if families did not want a social work referral. For example, Clover (SLP) shared, “Sometimes social work would be the obvious referral but a lot of families are really hesitant about social work, or they've had negative experiences with social work. They think of it more like…almost like they're spying on them.”

Another challenge related to families’ backgrounds was providing TIC to families who were linguistic minorities. Working with an interpreter added an extra layer that made TIC more difficult for providers. For example, Terry (OT) explained, “Language is a big barrier. My ability to connect with them is a little more separated. Because I have to talk, and the interpreter interprets, and a little something is lost there in that connection.”

Finally, participants reported that some families used practices that they did not agree with, which made it difficult to implement TIC. For example, Sarah (SLP) explained how a barrier to TIC was families’ “non‐receptiveness.” She went on to give an example:

So one family that I worked with, the mom was very non‐responsive to understand that her daughter's behaviors were telling her she didn't want the spoon shoved in her mouth… I actually ended up not continuing a service with this family because of that. That mom was force feeding the child. But she didn't, was not receptive to being coached, or being parent educated, or acknowledging those clear signs that that child was refusing food, as it's not healthy to make her do it. And so, that was a conflict. So that was a barrier to providing trauma informed care.

Similarly, Kylan (PT) gave an example of how a family's discipline practices were a barrier:

Grandma's trying to parent this baby, so she was very punishment oriented and really behavior focused. And so it was a challenge for everybody, particularly the social worker, I think, it mostly fell on her. But for all of us to try to support and respect this grandma and try to see if we could figure out some other strategies for her rather than just discipline and negative kinds of things.

Additionally, some participants recognized that these conflicting beliefs about practices were sometimes reflective of cultural differences. For example, Elizabeth (SW/DT) explained:

Sometimes trying to take into consideration certain cultures are may be a little bit harsher with their discipline, so trying to find that middle ground between teaching these families to be mindful of, maybe what has happened to these kiddos, but also being respectful of the way that they kind of do things. But that's a difficult one, because being mindful of a child's trauma does not always line up with somebody's parenting style.

Importantly, while the three previous examples each illustrated disagreement between families and providers about practices, it is notable that some providers, like Sarah, expected families to align with providers’ beliefs, while others, like Elizabeth, were more concerned with understanding how they could meet the family where they were.

### 3.4 Microsystem

The microsystem is composed of the direct influences on a child, including their environment, caregivers, and EI providers. We identified one theme within the microsystem that made it difficult for providers to use TIC: EI providers’ personal well‐being and comfort.

#### 3.4.1 EI providers’ personal well‐being and comfort

EI providers described threats to both their physical and emotional sense of safety when attempting to provide TIC in EI. These challenges were exacerbated by the requirement that EI services be provided in the child's natural settings, which is oftentimes the home. “When you go into people's homes it's so personal, it's so intimate…and the boundaries get kind of blurry.” In this quote, Kylan (PT) highlights how the deeply personal nature of home‐based service provision in EI creates unique challenges for providers. Participants noted several types of personal barriers to engaging in effective TIC, including their sense of physical safety and the emotional weight of the work.

When discussing barriers related to personal safety, some providers mentioned concerns about working in certain neighborhoods or homes. Charlotte (SLP) acknowledged that it can be hard to get providers to do an evaluation or pick up families in neighborhoods that are perceived as less safe. Charlotte herself has chosen not to serve families in those areas in an effort to prioritize her safety. She explained, “I don't wanna jeopardize what I perceive as my safety to go to a speech therapy session.” While describing this dilemma, she acknowledged how it creates access barriers for families living in certain regions saying, “These families are sitting and waiting for a therapist, or they can only get a video option and that's not working. And then they feel like they're stuck, and they have to make this work.” Terry (OT) discussed safety concerns more specifically within families’ homes:

I think safety is always always a barrier. Sometimes you just don't feel safe going somewhere. Or you go into a house‐ and I am not a big dog person. If there's big dogs, I'm scared. And if I personally pull up to a house that I don't like, I take a picture of the house and the house number, and I send it to somebody. You don't hear from me in an hour, this is where I am.

In addition to having to confront specific individual fears (e.g., dogs), Terry's response echoes concerns from other providers about feeling unsafe in certain homes without naming specific characteristics that made a home seem unsafe. Terry's approach was to have a personal safety communication plan, rather than avoid those homes. However, her approach may raise ethical considerations and may be based in implicit biases about families.

The emotional toll of serving children and families experiencing trauma was also cited as a common challenge. Melissa (DT) shared:

If there's a child who's in the foster system, there may be so much abuse that happened to that child that a therapist just can't personally deal with knowing what happened. Certain situations are very sad and heartbreaking and I think sometimes it's just too much for a therapist to have to deal with that.

Charlotte (SLP) exemplified the impact of this by sharing how she sits “at home worrying about families fairly often.” Similarly, Shirley (SLP) said, “I'm a little raw about it at the moment,” when discussing a recent case she found difficult. Kylan (PT) discussed feeling professionally unprepared for how to manage exposure to these types of emotionally challenging experiences:

Sometimes I feel like I'm just out there kind of absorbing a lot and I'm not sure exactly what to do with it. As a physical therapist I don't really know what to do with some of the feelings, the big experiences and feelings and things that families have been through that they share with me.

Clover (SLP) echoed similar observations of her peers. She noticed that many of them “have a hard time taking care of themselves, of holding onto some of these things and not having space to let them out.”

In response to the emotional toll that participants faced when using TIC, participants identified ways to manage it, such as having boundaries, engaging in self‐care, and debriefing with colleagues. Lucia (SLP) discussed her journey of developing boundaries with families:

I've also had to learn how to not take it home at night. Because as much as I care, I try to not answer phone calls at one o'clock in the morning. Not give rent money. And I think it takes a seasoned professional to have their own boundaries in how much you can help care for somebody who's been through trauma.

These boundaries spanned both interactions with the families (e.g., not giving money), but also boundaries for herself around acknowledging how much she can help. Clover (SLP) discussed the need for self‐care and self‐reflection in order to engage in effective TIC:

You have to do self‐care, so that you can be happy. But I don't think we really talk about, “What is my body doing when someone shares trauma? And why am I doing that? And what does that trigger? Where's my mind going? Am I thinking about something else? Am I focused on what they're saying? How can we stay focused on what they're saying?”

She suggested that self‐reflective work would be valuable professional development. Emma (SLP) focused more on the need for an appropriate space to debrief about TIC:

Sometimes you can receive emotional support and give emotional support to those providers. Some of these kids are very emotionally taxing but having a team, especially when it's a team of people I know, like a good [Individualized Family Service Plan] team… that's what I'm looking for. That's a provider I'm gonna use as a source of support for me when providing this care cause they're gonna get it.

She compared this kind of professional support to her husband who is also supportive but “doesn't understand it” which makes him a less effective source of support in this instance. She needed someone who “gets it” and who she trusts. In conclusion, EI providers perceive threats to their physical and emotional safety as barriers to their ability to provide effective TIC. To address these threats, they expressed needing individual plans (e.g., self‐care plans) and opportunities to lean on peers and professional networks.

## 4 DISCUSSION

One of our most prevalent findings was related to the role of EI providers in using TIC with families, an issue that rose across multiple social ecological systems (Bronfenbrenner, 1977). Notably, the data illustrates how macrosystem issues trickle down and impact the smaller systems. For example, at the macrosystem level, participants were not sure how TIC fit into EI or specific EI disciplines, citing a lack of clarity in the EI system. With a lack of role clarity related to TIC in EI, it left EI providers without direction on how to implement the practice. This was evident in our findings as participants discussed the exosystem barrier of not receiving training support to implement TIC, especially within the context of their specific discipline. Given this lack of exosystem support, we found that participants had varying beliefs about *who* should implement TIC, with some believing it should be reserved for the social work discipline, while others believed everyone should implement TIC. These varied beliefs impacted the ways in which providers interacted with one another and families, such as some providers believing that information about trauma should be withheld from others. Another challenge at the macrosystem level that impacted the smaller systems was the limited number of EI providers, especially those who served in the role of psychology/social work. This shortage led to several issues for participants. For example, one provider explained that DTs served as temporary replacements for SWs, a practice that is not recommended in the field (Division for Early Childhood, 2024). Another issue reported by participants stemming from the shortage showed up in mesosystem interactions, in which social work services were withheld or reserved for only the most severe circumstances. Noteworthy, while service coordinators and evaluators were often those discussed by participants as making these decisions in response to the provider shortage, we encourage readers to focus on how the EI system as a whole can strengthen their policies and procedures to prevent this from happening, rather than blaming any specific individuals or roles.

Another important point of discussion is how providers’ assumptions, beliefs, and biases emerged in this study as they discussed trauma and TIC. For example, some providers had assumptions about the safety of families’ homes, resulting in them either not serving families in areas they perceived as less safe or using questionable practices (e.g., taking a photo of a house) to ensure their safety. These findings contribute to the systemic bias that we already know exists, such as marginalized families experiencing disparities in access to screening and/or EI services (Blanchard et al., 2021; Keating et al., 2022; McManus et al., 2020; Moran & Sheppard, 2022). Another area where providers' beliefs and biases surfaced was in the way they described certain family dynamics. For example, providers discussed challenges with providing TIC when they encountered language or cultural barriers. Some felt that the relational practices inherent to TIC in EI were undermined by communicating through an interpreter. They also struggled when they encountered parenting practices that they perceived as misaligned with TIC. A few participants were even aware that their thoughts about families might be judgmental or biased, yet still expressed not knowing how to proceed. Thus, we emphasize that the barrier to TIC is not the family's practices, background, or beliefs, but rather, providers' inability and lack of systemic support to navigate those differences in a trauma‐informed way.

Finally, the participants who shared their experience of attending a training on TIC expressed dissatisfaction with the sessions. They found them either too introductory or not applicable enough to EI practice. This indicates a need for more comprehensive training in TIC that directly relates to EI providers. This could take the form of discipline‐specific training, allowing providers to learn specific practices to use with children and families (e.g., SLPs learning about trauma‐informed feeding practices, PTs learning about the importance of respecting child autonomy during positioning). It may also be helpful to have a unified training where EI providers from all disciplines come together to ensure a cohesive understanding of TIC. Importantly, these trainings should embed the components suggested by participants, such as self‐ and group‐reflection opportunities and incorporation of lived experiences. Additionally, we can apply what makes trauma training successful according to education professionals, including the importance of video demonstration, reflection, practice opportunities, and group discussion (Goldenthal et al., 2024), as well as developing action plans (Smith et al., 2025).

### 4.1 Implications for policy and personnel preparation

The findings related to EI providers’ roles in providing TIC signify a dire need for clarity in the field. This should begin with a top‐down approach, in which the EI system itself takes a stand on TIC in EI, as recommended by others (e.g., Munger et al., 2023). Thus, statewide EI systems need to develop policies about the use of TIC in EI, such as who on the team should be informed of trauma, whether or not social work should be an automatic referral for families who report trauma, the roles and responsibilities of all disciplines in responding to trauma, and how to navigate perceived tensions between TIC and families’ beliefs/practices. With these policies in place, EI system leaders can then allocate targeted exosystem resources (e.g., training, supervision) for EI providers to implement TIC within the context of their role.

Further, individuals who prepare and provide ongoing training to EI providers should consider embedding content on TIC into existing coursework and training. One notable option in pre‐service preparation is to develop interdisciplinary trauma‐focused training programs or certificates so that prospective providers can learn alongside those with different disciplines and thus increase their understanding of their own and one another's roles in providing TIC in EI. Moreover, training for pre‐service and in‐service EI providers should prepare them to navigate the complex interactions they will encounter in the field related to trauma, such as gatekeeping and family–provider interactions. Importantly, EI providers need opportunities to learn about how systemic and individual biases impact families in EI (Blanchard et al., 2021). Recognizing how the system makes it more difficult to support trauma‐impacted families is a first step to breaking down barriers. Finally, the EI system can implement reflective supervision, a relationship‐focused supervisory process that supports professionals in their ability to reflect on their practice and address any emotional responses that occur in practice (Tobin et al., 2023). Reflective supervision can increase reflective capacity, prevent burnout, and promote work satisfaction (University of Minnesota, Center for Early Education & Development, 2019; Watson et al., 2016). Thus, EI leaders may consider implementing reflective supervision as a means to support EI providers as they work with children and families who have experienced trauma.

### 4.2 Implications for practice

There are three major implications from this study related to how providers implement TIC. First, while the EI system should prepare and support providers to acknowledge bias, providers themselves must also address their own assumptions and implicit biases about families and about trauma. Strategies for doing this include recognizing and replacing biased thoughts about families, taking on the perspective of the family member/child, and intentionally regarding families in a positive manner (Devine et al., 2012; Venet, 2021). The second implication for EI providers is to seek clarification about what TIC can look like within their discipline. There are resources available from discipline‐specific organizations (e.g., American Physical Therapy Association, American Occupational Therapy Association, American Speech–Language‐Hearing Association, Council for Exceptional Children) that discuss TIC. While these may not focus on EI specifically, they can serve as a starting point for providers. Finally, EI providers need to ensure they attend to their own well‐being. It is common for help‐giving professionals to experience compassion fatigue, in which they have a physical or emotional reaction to someone else's trauma (Nicholson et al., 2023), as indicated by our participants. Participants in this study also showed signs of moral distress (i.e., discomfort that arises when one knows the right thing to do but institutional constraints make it difficult to pursue the right course of action; Jameton (1984). Feelings of moral distress and compassion fatigue can lead to burnout in professionals (Maunder et al., 2023). Thus, it is critical for providers to engage in self‐care, mindfulness, and reflection, as well as identify social supports to reduce the impact of secondary trauma on themselves and have access to resources for navigating these feelings, such as reflective supervision as discussed earlier.

### 4.3 Limitations and future directions for research

First, though we suspect there are similar issues across US states, all participants in this study worked in Illinois. Each state has different procedures for EI and is situated in a different context, therefore, the findings may be less transferable to EI providers in other states. Likewise, it is possible that some of our findings do not generalize to EI systems in other countries. Thus, we recommend that readers reflect on whether the findings identified in this study align with issues in their specific context and attend to implications that best meet their needs. Another limitation is that we only sought the perspective of EI providers that provide direct services; service coordinators and family members will have additional perspectives on the use of TIC, which are worth exploring. Future research can further explore the roles of various EI disciplines in implementing TIC. This could be investigated using a large‐sample survey with EI providers from multiple disciplines across the United States. Further, given that this study relied only on provider reports, observational studies could provide insight into the current state of TIC in the field, including the roles of various disciplines, strengths and barriers, and families’ reactions to TIC. Another area of research could be designing and piloting a training about TIC for EI providers with pre‐ and post‐data collection to determine the training's effectiveness.

Another limitation is that participants self‐selected to participate in this study and had a high average number of years of experience (15.4 years). This means that participants were likely not new to TIC, had more experience in EI, and had a deeper understanding of barriers and needs related to the use of TIC. Newer EI providers may experience additional barriers that were not captured. Finally, some of the biased views about families emerged because of the questions we asked and maybe even due to participants’ comfort speaking transparently with us. We did not ask participants to expand on their biased remarks, and thus, our interpretation of them is limited. Researchers must continually reflect on how to ask EI providers about challenges, as it inherently opens the door for critiques about families. They also should consider what their role is in responding, disrupting, and/or digging deeper to understand such comments.

## 5 CONCLUSION

The need for TIC in EI is a growing concern in the field. This qualitative study illustrated the barriers and needs related to TIC in EI across multiple social ecological systems (Bronfenbrenner, 1977). This approach can help EI systems identify where the barriers are and how to address them. Notably, this study highlights a need for both system and individual‐level supports to enhance TIC in EI for families.

## CONFLICT OF INTEREST STATEMENT

The authors have no known conflicts of interest.
